# Inflammatory, Oxidative Stress, and Angiogenic Growth Factor Responses to Repeated-Sprint Exercise in Hypoxia

**DOI:** 10.3389/fphys.2019.00844

**Published:** 2019-08-09

**Authors:** Nobukazu Kasai, Chihiro Kojima, Daichi Sumi, Akiho Ikutomo, Kazushige Goto

**Affiliations:** ^1^Graduate School of Sport and Health Science, Ritsumeikan University, Kusatsu, Japan; ^2^Japan Society for the Promotion of Science, Tokyo, Japan; ^3^Faculty of Sport and Health Science, Ritsumeikan University, Kusatsu, Japan

**Keywords:** maximal sprint, hypoxic exercise, inflammation, angiogenic growth factor, track and field sprinters

## Abstract

The present study was designed to determine the effects of repeated-sprint exercise in moderate hypoxia on inflammatory, muscle damage, oxidative stress, and angiogenic growth factor responses among athletes. Ten male college track and field sprinters [mean ± standard error (SE): age, 20.9 ± 0.1 years; height, 175.7 ± 1.9 cm; body weight, 67.3 ± 2.0 kg] performed two exercise trials in either hypoxia [HYPO; fraction of inspired oxygen (F_i_O_2_), 14.5%] or normoxia (NOR; F_i_O_2_, 20.9%). The exercise consisted of three sets of 5 s × 6 s maximal sprints with 30 s rest periods between sprints and 10 min rest periods between sets. After completing the exercise, subjects remained in the chamber for 3 h under the prescribed oxygen concentration (hypoxia or normoxia). The average power output during exercise did not differ significantly between trials (*p* = 0.17). Blood lactate concentrations after exercise were significantly higher in the HYPO trial than in the NOR trial (*p* < 0.05). Plasma interleukin-6 concentrations increased significantly after exercise (*p* < 0.01), but there was no significant difference between the two trials (*p* = 0.07). Post-exercise plasma interleukin-1 receptor antagonist, serum myoglobin, serum lipid peroxidation, plasma vascular endothelial growth factor (VEGF), and urine 8-hydroxydeoxyguanosine concentrations did not differ significantly between the two trials (*p* > 0.05). In conclusion, exercise-induced inflammatory, muscle damage, oxidative stress, and VEGF responses following repeated-sprint exercise were not different between hypoxia and normoxia.

## Introduction

Repeated-sprint training in hypoxia (RSH), consisting of a brief duration of maximal sprints (≤30 s) interspersed with partial recoveries, has become prevalent among various types of athletes (Galvin et al., 2013; Faiss et al., 2013a,b, 2015; Gatterer et al., 2014, 2019; Brocherie et al., 2015, 2017; Kasai et al., 2015, 2017; Hamlin et al., 2017; Girard et al., 2017a). RSH is one of the potent training procedures for improvement of repeated-sprint ability among team sport athletes (Girard et al., 2011; Billaut et al., 2012). Moreover, recommended procedures for RSH were proposed in a recent review (Brocherie et al., 2017). Particularly, well-known in this regard, Faiss et al. (2013a,b) demonstrated that RSH for 4 weeks significantly improved the number of sprints until fatigue during repeated-sprint test (repeated 10 s maximal sprint with 20 s active recovery until exhaustion) among well-trained cyclists. Furthermore, RSH for 3 weeks significantly improved repeated-sprint ability (8 m × 20 m sprint with 20 s rest between sprints) among rugby players (Hamlin et al., 2017).

Several physiological mechanisms underlying the further improvement of repeated-sprint ability following RSH have been suggested, including augmented blood perfusion (Faiss et al., 2013a, 2015), increased glycolytic enzymes (Faiss et al., 2013a), and augmented muscle glycogen and phosphocreatine (PCr) contents (Kasai et al., 2017, 2019). Sprint training in hypoxia (e.g., submaximal sprint exercise or high-intensity sprint exercise) improved mitochondrial function (Geiser et al., 2001), muscle buffering capacity (Mizuno et al., 1990), and phosphofructokinase activity (Puype et al., 2013). Moreover, repeated-sprint ability is associated with PCr resynthesis capacity between sprints (Girard et al., 2011; Billaut et al., 2012; Mendez-Villanueva et al., 2012) and it is influenced by aerobic capacity (Bishop and Edge, 2006). Therefore, enhanced aerobic capacity following RSH may also improve repeated-sprint ability. Furthermore, because hypoxia stimulates vascular endothelial growth factor (VEGF) production, which promotes angiogenic adaptation (Egginton, 2009), it is plausible that increased VEGF concentration due to sprint exercise in hypoxia promotes capillary density, leading to improved oxygen supply capacity for working muscles. However, the acute VEGF response to repeated-sprint exercise in hypoxia has not yet been elucidated.

Although a number of previous studies have reported the efficacy of RSH, we are aware that any putative negative impact of RSH has not been examined. To our knowledge, only one previous study investigated inflammatory and oxidative stress responses to acute repeated-sprint exercise in moderate hypoxia [three successive sets of 9 s × 5 s maximal sprints in hypoxia (F_i_O_2_, 14.5%)] (Goods et al., 2016). Consequently, no apparent differences in exercise-induced elevations of interleukin-6 (IL-6, an indicator of the inflammatory response) or F_2_-isoprostane (an indicator of oxidative stress) were observed between hypoxia and normoxia. However, caution is necessary in this interpretation because the duration of exposure to hypoxia during exercise was limited (approximately 16 min). During high-intensity exercise in hypoxia, anaerobic energy supply (i.e., the glycolytic pathway) is augmented (Ogura et al., 2006). Because lower muscle glycogen promotes IL-6 production in skeletal muscle (Steensberg et al., 2001), and exercise-induced IL-6 elevation may be augmented following repeated-sprint exercise in hypoxia. Augmented IL-6 stimulates the production of interleukin-1 receptor antagonist (IL-1ra), which plays an anti-inflammatory role, during the early phase of post-exercise (Nehlsen-Cannarella et al., 1997; Horn et al., 2000). Moreover, hypoxia itself augmented reactive oxygen species (ROS), resulting in aggravated oxidative stress (Dosek et al., 2007; Quindry et al., 2016). In addition, it is important to determine the exercise-induced muscle damage response following RSH, because high-intensity exercise also increases myoglobin (Mb, an indicator of the muscle damage response) (Naclerio et al., 2014). However, inflammatory, oxidative stress, and muscle damage responses following acute repeated-sprint exercise in moderate hypoxia have not been fully investigated. This information would help for developing appropriate training program of RSH with a lower risk of overtraining.

Therefore, the purpose of the present study was to determine the effects of acute repeated-sprint exercise in moderate hypoxia on inflammatory, muscle damage, oxidative stress, and angiogenic growth factor responses among competitive athletes. We hypothesized that acute repeated-sprint exercise in moderate hypoxia would augment post-exercise inflammatory, oxidative stress, and angiogenic growth factor responses compared with those following the same exercise conducted in normoxia.

## Materials and Methods

### Subjects

Ten male college track and field sprinters (100–400 m) participated in the study [mean ± standard error (SE): age, 20.9 ± 0.1 years; height, 175.7 ± 1.9 cm; body weight (BW), 67.3 ± 2.0 kg; body mass index (BMI), 21.7 ± 0.7 kg/m^2^; athletic career, 8.1 ± 0.5 years; 100 m personal best, 10.99 ± 0.12 s]. All athletes were born at and currently living at sea level. They belonged to the track and field club at the same university and maintained training for 5 days/week (3 h/day). The present study was conducted during the basic training phase in a periodized training program. All subjects were informed about the risks and benefits of the present study and provided written informed consent. The present study was approved by the Ethics Committee for Human Experiments at Ritsumeikan University and was conducted in accordance with the Declaration of Helsinki.

### Experimental Design

All subjects visited the laboratory three times throughout the experiment. On the first visit, a familiarization trial for the exercise tests was performed. Approximately 1 week after the familiarization session, subjects visited the laboratory twice (main trials) on different days. Two main trials were separated by at least 1 week to eliminate the influence of the previous session. On the days for the main trials, subjects visited the laboratory in the morning (8:00) following an overnight fast (at least 10 h). All subjects were asked to abstain from alcohol, caffeine, dietary supplements, antioxidant material, and strenuous exercise for at least 48 h before each trial. In addition, dietary content during three meals (breakfast, lunch, and dinner) on the day before the trial was matched between trials.

Subjects conducted two exercise trials in hypoxia [HYPO; fraction of inspired oxygen (F_i_O_2_), 14.5%, equivalent to a simulated altitude of 3,000 m] or normoxia (NOR; F_i_O_2_, 20.9%) in a randomized order. After completing the exercise, subjects remained in the chamber for 3 h under the prescribed oxygen concentration. The study followed a single-blinded, placebo-controlled design. Because previous studies have suggested that the placebo effect may be at least partially responsible for performance changes in hypoxia (Lundby et al., 2012; Inness et al., 2016), subjects were not given any information about the selected condition. The questionnaire revealed that 50% of subjects answered correctly about the order of the trial, in a support of the blinding procedure. Both trials were completed in an environmentally controlled chamber. In the present study, a whole-room type (14.8 m^2^) hypoxic chamber was used, and hypoxic conditions were created by insufflating nitrogen as reported previously (Morishima et al., 2014; Kasai et al., 2015; Goto et al., 2018; Kojima et al., 2019). Temperature and humidity in the chamber were automatically maintained at 23°C and 50%, respectively. Oxygen and carbon dioxide concentrations within the chamber were continuously monitored.

During the two main experiments, changes in repeated-sprint ability, percutaneous oxygen saturation (SpO_2_), heart rate (HR), and scores of subjective variables, blood variables, metabolic responses, and urine variables were collected and compared.

### Repeated-Sprint Exercise

Following an approximately 20 min rest period (completing baseline measurements, including respiratory and blood measurements) after entering the chamber, subjects began a standardized warm-up consisting of 5 min of cycling at 80 W, followed by 2 s × 3 s maximal sprints in which the load was equivalent to 7.5% of BW. At 5 min after warm-up, they conducted repeated-sprint exercises (three sets of 5 s × 6 s maximal sprints with 30 s rest periods between sprints and 10 min rest periods between sets) using an electromagnetically braked cycle ergometer (Power Max VIII; Konami Corp., Tokyo, Japan). The applied load was equivalent to 7.5% of BW. After completing the final sprint, the subjects remained in the chamber for 3 h. Therefore, the total duration of exposure to hypoxia (HYPO) or normoxia (NOR) was approximately 4 h. Peak and mean power output during each sprint was recorded. Average power output was calculated as an averaged mean power output throughout each set of sprints (5 sprints for each set). The power output decrement during repeated-sprint exercise was calculated [100 × (total power output)/ideal power output (the number of sprints × highest power output)] − 100 (Glaister et al., 2008). Subjects were verbally encouraged throughout each sprint to perform the exercise with maximal effort.

### SpO_2_, HR, and Subjective Variables

SpO_2_ was monitored every second using a finger pulse oximeter placed on the tip of the right forefinger (Pulsox-Me300, Teijin Co., Ltd., Tokyo, Japan) during exercise and rest period. HR was also measured continuously during exercise and rest period using a wireless HR monitor (RS400, Polar Electro, Tokyo, Japan). Scores of fatigue for respiration and lower-limb muscles were evaluated using a 100-mm visual analog scale (Saanijoki et al., 2015) before exercise, immediately after each set of exercise, and every 30 min during the 3 h post-exercise period.

### Blood Variables

Venous blood samples were collected from the antecubital vein on four occasions: before exercise (after 20 min of rest in the chamber), immediately after exercise, and 1 and 3 h after exercise. All blood samples were taken while the subjects were sitting in a chair. Serum and plasma samples were obtained after a 10 min centrifugation at 4°C and stored at −80°C until analysis. Blood glucose, lactate, serum myoglobin (Mb), plasma IL-6, IL-1ra, serum lipid peroxidation (LPO), and plasma VEGF concentrations were measured. Blood glucose and lactate concentrations were measured using a glucose analyzer (Free style, Nipro Co., Osaka, Japan) and a lactate analyzer (Lactate Pro, Arkray Co., Kyoto, Japan) immediately after blood collection, respectively. Blood glucose concentration was measured in duplicate, and the average value was used for analysis. The intraclass correlation coefficient (ICC) between measurements for this analysis was 0.99. Serum Mb concentration was measured using a double-antibody radioimmunoassay (RIA), and serum LPO concentration was measured using a thiobarbituric acid (TBA) method at a clinical laboratory (Nikken Seil Co., Ltd., Shizuoka, Japan). Plasma IL-6, IL-1ra, and VEGF concentrations were assayed with an ELISA Kit (R&D Systems, Minneapolis, MN, USA). The intra-assay coefficient of variation (CV) for each measurement was as follows: 2.7% for Mb, 4.3% for IL-6, 4.7% for IL-1ra, 4.8% for LPO, and 6.3% for VEGF.

### Metabolic Responses

Respiratory variables were evaluated before exercise, during exercise, and during a 3 h post-exercise period (samples were collected every 30 min during 3 h of post-exercise). Respiratory samples were collected by the breath-by-breath method and analyzed using an automatic gas analyzer (AE300S; Minato Medical Science Co., Ltd., Tokyo, Japan). Oxygen uptake (${\dot{V}O}_{2}$), carbon dioxide production (${\dot{V}\mathrm{CO}}_{2}$), and ventilation volume ($\dot{V}E$) were measured. The respiratory exchange ratio (RER) was determined from the ${\dot{V}O}_{2}$ and ${\dot{V}\mathrm{CO}}_{2}$ values (${\dot{V}\mathrm{CO}}_{2}\;/\;{\dot{V}O}_{2}$). The collected data were averaged every 6 s during exercise and every 30 s during rest (before exercise) and post-exercise. Energy expenditure was calculated using the following equation (Weir, 1949):

$$\begin{array}{l} \mathrm{Energy expenditure}\;\left(\mathrm{kcal}\;/\;\min\right)\;\;=\;\;3.9\;\;\times \;\;{\dot{V}O}_{2}\;\left(L\;/\;\min\right)\\ \;\;\;\;\;\;\;\;\;\;\;\;\;\;\;\;\;\;\;\;\;\;\;\;\;\;\;\;\;\;\;\;\;\;\;\;\;\;\;\;\;\;\;\;\;\;\;\;\;\;\;\;\;\;+\;\;1.1\;\;\times \;\;{\dot{V}\mathrm{CO}}_{2}\;\left(L\;/\;\min\right). \end{array}$$

### Urine 8-OHdG Excretion

Urine was collected during the post-exercise period. The collected urine was stored at −80°C until analysis. From collected urine samples, urine 8-hydroxydeoxyguanosine (8-OHdG) excretion (calculated by urine 8-OHdG concentration × urine volume) during 3 h of post-exercise was measured using high-performance liquid chromatography at a clinical laboratory (SRL Inc., Tokyo, Japan). The intra-assay CV for 8-OHdG was 1.8%.

### Statistical Analysis

All data are expressed as the mean ± SE. Two-way repeated-measure analysis of variance (ANOVA) was applied to assess the interaction (time × trial) and main effects (trial and time). When ANOVA revealed a significant interaction or main effect, the Tukey-Kramer test was used to identify differences. Average SpO_2_, HR values, peak power output, and urine 8-OHdG excretion were compared between the two trials using a paired *t*-test. A *p* < 0.05 was considered significant for all measurements. Effect size was calculated by partial eta squared ($\eta_{p}^{2}$) for two-way ANOVA with repeated measures and Cohen’s d (*d*) for a paired *t*-test.

## Results

### SpO_2_ and HR

The average SpO_2_ values during exercise were significantly lower in the HYPO trial (86.5 ± 0.8%) compared with the NOR trial (95.1 ± 0.4%, *p* < 0.01, *d* = 4.25). Similarly, SpO_2_ remained significantly lower in the HYPO trial (89.4 ± 0.4%) than in the NOR trial (96.3 ± 0.2%, *p* < 0.01, *d* = 8.13) during 3 h of post-exercise. The average HR tended to be higher in the HYPO trial than in the NOR trial during exercise (including rest periods between sprints; HYPO, 144 ± 2 bpm; NOR, 139 ± 2 bpm; *p* = 0.05, *d* = 0.75) and post-exercise (HYPO, 89 ± 2 bpm; NOR, 82 ± 3 bpm; *p* = 0.01, *d* = 0.90).

### Power Output During Repeated-Sprint Exercise

**Figure 1:**
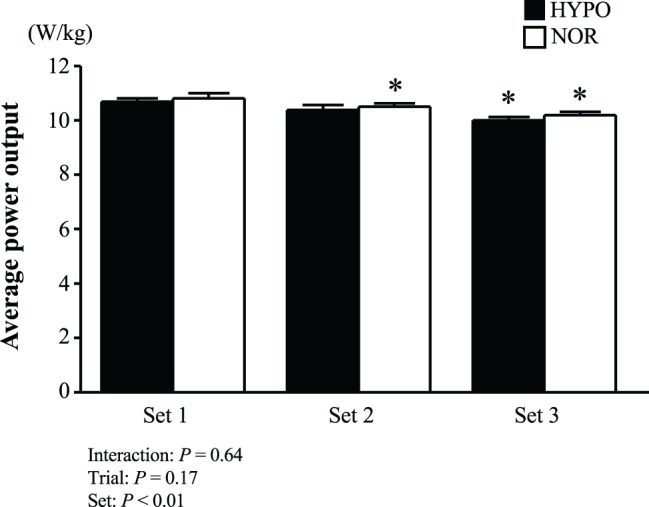
Average power output during each set. Power outputs of five sprints during each set were averaged. Values are means ± SE. ^*^p < 0.05 vs. Set 1.

The peak power output did not differ significantly between the HYPO (14.3 ± 0.2 W/kg) and NOR (14.2 ± 0.2 W/kg, *p* = 0.17, *d* = 0.18) trials. Figure 1 shows the average power output in each set during repeated-sprint exercise (three sets of 5 s × 6 s sprints). Average power output decreased with the progress of sets (main effect for set: *p* < 0.01, $\eta_{p}^{2}$ = 0.74). However, no significant interaction between trial and set (*p* = 0.64, $\eta_{p}^{2}$ = 0.04) or main effect for trial (*p* = 0.17, $\eta_{p}^{2}$ = 0.20) was observed. The power output decrement during each set showed significant main effects for set (*p* = 0.02, $\eta_{p}^{2}$ = 0.38) and trial (*p* < 0.01, $\eta_{p}^{2}$ = 0.62), but no significant interaction between trial and set was observed (*p* = 0.78, $\eta_{p}^{2}$ = 0.02). The power output decrement in the HYPO trial was profound, differing significantly from the NOR trial during set 3 (HYPO, 12.7 ± 0.2%; NOR, 9.8 ± 0.1%, *p* < 0.05).

### Blood Variables

Table 1 shows blood glucose and lactate concentrations. Blood glucose concentration increased markedly immediately after exercise in both trials (main effect for time: *p* < 0.01, $\eta_{p}^{2}$ = 0.82). However, no significant interaction between time and trial (*p* = 0.51, $\eta_{p}^{2}$ = 0.08) or main effect for trial (*p* = 0.18, $\eta_{p}^{2}$ = 0.19) was observed. Both trials showed marked increases in blood lactate concentrations (main effect for time: *p* < 0.01, $\eta_{p}^{2}$ = 0.99). Furthermore, there was a significant interaction between time and trial (*p* < 0.01, $\eta_{p}^{2}$ = 0.69) and a main effect for trial (*p* < 0.01, $\eta_{p}^{2}$ = 0.83). Blood lactate concentrations were significantly higher in the HYPO trial both immediately and 60 min after exercise (*p* < 0.05 each).

**Table 1:** Blood glucose and lactate concentrations.

		Pre	0 min	60 min	180 min	Interaction ($\eta_{p}^{2}$)
						Time, Trial ($\eta_{p}^{2}$)
Glucose (mg/dl)	HYPO	88 ± 2	112 ± 5^*^	73 ± 2^*^	83 ± 2	*p* = 0.51 (0.08)
	NOR	88 ± 2	114 ± 7^*^	77 ± 3^*^	89 ± 3	*p* < 0.01 (0.82), *p* = 0.18 (0.19)
Lactate (mmol/L)	HYPO	1.6 ± 0.1	22.5 ± 0.9^*^^,^^†^	4.9 ± 0.1^*^^,^^†^	2.0 ± 0.1	*p* < 0.01 (0.69)
	NOR	1.5 ± 0.1	19.3 ± 0.7^*^	4.2 ± 0.3^*^	2.0 ± 0.1	*p* < 0.01 (0.99), *p* < 0.01 (0.83)

*Values are shown as mean ± SE*.

* *p < 0.05 vs. Pre*.

† *p < 0.05 vs. NOR trial*.

**Figure 2:**
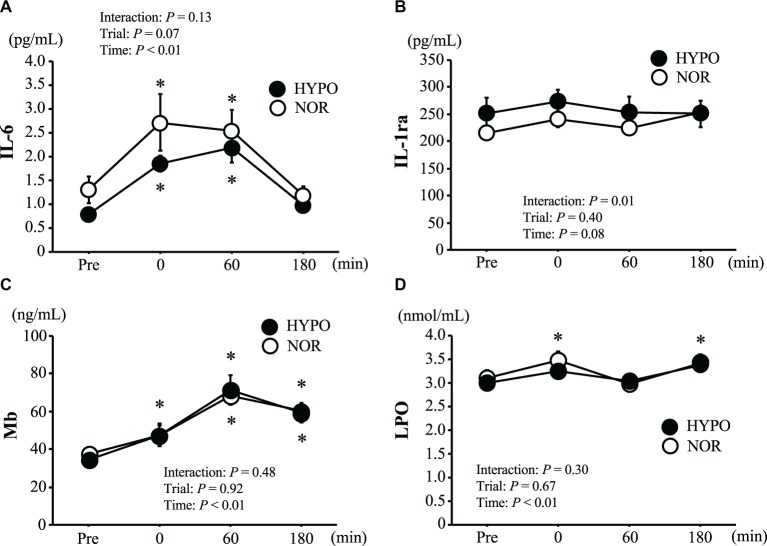
Changes in plasma interleukin-6 (IL-6) **(A)**, and plasma interleukin-1 receptor antagonist (IL-1ra) **(B)**, serum myoglobin (Mb) **(C)** and serum lipid peroxidation (LPO) **(D)**. Values are means ± SE. ^*^p < 0.05 vs. Pre.

Figure 2 shows the changes in plasma IL-6, IL-1ra, serum Mb, and LPO concentrations. The plasma IL-6 concentration increased significantly after exercise (main effect for time: *p* < 0.01, $\eta_{p}^{2}$ = 0.64), whereas there was no significant interaction between time and trial (*p* = 0.13, $\eta_{p}^{2}$ = 0.18) or a main effect for trial (*p* = 0.07, $\eta_{p}^{2}$ = 0.32). The plasma IL-1ra concentration showed a significant interaction between time and trial (*p* = 0.01, $\eta_{p}^{2}$ = 0.36), but no significant main effect for time (*p* = 0.08, $\eta_{p}^{2}$ = 0.27) or trial (*p* = 0.40, $\eta_{p}^{2}$ = 0.09) was observed. The serum Mb concentration was significantly elevated after exercise (main effect for time: *p* < 0.01, $\eta_{p}^{2}$ = 0.84). However, no significant interaction between time and trial (*p* = 0.48, $\eta_{p}^{2}$ = 0.08) or main effect for trial (*p* = 0.92, $\eta_{p}^{2}$ = 0.01) was observed. A significant increase in serum LPO concentration was observed only in the NOR trial (*p* < 0.05). However, there was no significant interaction between time and trial (*p* = 0.30, $\eta_{p}^{2}$ = 0.13) or a main effect for trial (*p* = 0.67, $\eta_{p}^{2}$ = 0.02).

**Figure 3:**
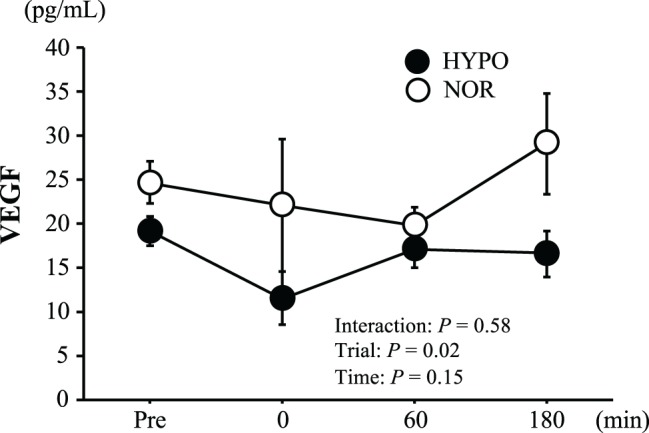
Changes in plasma vascular endothelial growth factor (VEGF). Values are means ± SE.

Figure 3 shows changes in plasma VEGF concentrations. There was no significant interaction between trial and time (*p* = 0.58, $\eta_{p}^{2}$ = 0.07) or a main effect for time (*p* = 0.15, $\eta_{p}^{2}$ = 0.18). Moreover, in spite of a significant main effect for trial (*p* = 0.02, $\eta_{p}^{2}$ = 0.30), there was no significant difference between the trials at any point.

### Metabolic Responses

Table 2 shows the changes in ${\dot{V}O}_{2}$, ${\dot{V}\mathrm{CO}}_{2}$, and $\dot{V}E$ during exercise. For ${\dot{V}O}_{2}$, there was a significant interaction between time and trial (*p* < 0.01, $\eta_{p}^{2}$ = 0.44) and main effects for time (*p* < 0.01, $\eta_{p}^{2}$ = 0.51) and trial (*p* < 0.01, $\eta_{p}^{2}$ = 0.93). ${\dot{V}\mathrm{CO}}_{2}$ showed significant main effects for time (*p* < 0.01, $\eta_{p}^{2}$ = 0.91) and trial (*p* < 0.01, $\eta_{p}^{2}$ = 0.72), but no significant interaction was detected (*p* = 0.30, $\eta_{p}^{2}$ = 0.13). Both ${\dot{V}O}_{2}$ and ${\dot{V}\mathrm{CO}}_{2}$ values were significantly lower in the HYPO trial than in the NOR trial during all sets (*p* < 0.05 for each). For $\dot{V}E$, there were significant main effects for time (*p* < 0.01, $\eta_{p}^{2}$ = 0.78) and trial (*p* < 0.01, $\eta_{p}^{2}$ = 0.81), whereas no significant interaction was observed (*p* = 0.32, $\eta_{p}^{2}$ = 0.12). $\dot{V}E$ was significantly higher in the HYPO trial than in the NOR trial during all sets (*p* < 0.05 each).

**Table 2:** Respiratory variables during exercise session.

		Set 1	Set 2	Set 3	Interaction ($\eta_{p}^{2}$)
					Time, Trial ($\eta_{p}^{2}$)
${\dot{V}O}_{2}$ (L)	HYPO	47.0 ± 1.5^†^	48.1 ± 1.8^†^	47.2 ± 1.9^†^	*p* < 0.01 (0.44)
	NOR	57.0 ± 1.7	60.0 ± 1.9^*^	59.7 ± 1.9^*^	*p* < 0.01 (0.51), *p* < 0.01 (0.93)
${\dot{V}\mathrm{CO}}_{2}$ (L)	HYPO	15.7 ± 0.6^†^	13.2 ± 0.4^*^^,^^†^	11.7 ± 0.4^*^^,^^†^	*p* = 0.30 (0.13)
	NOR	16.5 ± 0.6	14.0 ± 0.5^*^	13.0 ± 0.5^*^	*p* < 0.01 (0.91), *p* < 0.01 (0.72)
$\dot{V}E$ (L/min)	HYPO	107.3 ± 8.7^†^	120.3 ± 8.3^*^^,^^†^	121.1 ± 7.9^*^^,^^†^	*p* = 0.32 (0.12)
	NOR	94.7 ± 7.6	105.3 ± 7.7^*^	110.9 ± 8.1^*^	*p* < 0.01 (0.78), *p* < 0.01 (0.81)

*Values are shown as mean ± SE*.

*The ${\dot{V}O}_{2}$ and ${\dot{V}\mathrm{CO}}_{2}$ data during exercise and rest period between sprints were summed over the all sets, and $\dot{V}E$ was averaged in each set*.

* *p < 0.05 vs. set 1*.

† *p < 0.05 vs. NOR trial*.

Table 3 shows the changes in ${\dot{V}O}_{2}$, ${\dot{V}\mathrm{CO}}_{2}$, RER, $\dot{V}E$, and energy expenditure during the 3 h post-exercise period. There was a significant main effect of time for ${\dot{V}O}_{2}$ (*p* < 0.01, $\eta_{p}^{2}$ = 0.73) and ${\dot{V}\mathrm{CO}}_{2}$ (*p* < 0.01, $\eta_{p}^{2}$ = 0.39). ${\dot{V}O}_{2}$ remained significantly higher than the pre-exercise value in both trials (*p* < 0.05 each), except for 150 and 180 min after exercise in the NOR trial. For ${\dot{V}\mathrm{CO}}_{2}$, both trials showed significantly higher values than pre-exercise only at 30 min after exercise (*p* < 0.05). RER showed a significant main effect for time (*p* < 0.01, $\eta_{p}^{2}$ = 0.53). RER remained significantly lower in the HYPO trial (vs. the pre-exercise value) throughout the 3 h post-exercise period (*p* < 0.05). For $\dot{V}E$, there were significant main effects for time (*p* < 0.01, $\eta_{p}^{2}$ = 0.70) and trial (*p* < 0.01, $\eta_{p}^{2}$ = 0.67). These values were significantly higher in the HYPO trial at several points (*p* < 0.05 each). For energy expenditure, there was a significant main effect for time (*p* < 0.01, $\eta_{p}^{2}$ = 0.66), but no significant interaction (*p* = 0.64, $\eta_{p}^{2}$ = 0.07) or main effect for trial (*p* = 0.39, $\eta_{p}^{2}$ = 0.08) was observed. In the NOR trial, exercise-induced elevation of energy expenditure (vs. the pre-exercise value) disappeared within the initial 90 min of post-exercise. However, the HYPO trial showed significantly higher energy expenditure over the 3 h of post-exercise (*p* < 0.05).

**Table 3:** Respiratory variables and energy expenditure during post-exercise period.

		Pre	30 min	60 min	90 min	120 min	150 min	180 min	Interaction ($\eta_{p}^{2}$)
									Time, Trial ($\eta_{p}^{2}$)
${\dot{V}O}_{2}$ (ml/min)	HYPO	254 ± 9	340 ± 12^*^	296 ± 15^*^	289 ± 11^*^	285 ± 11^*^	290 ± 9^*^	282 ± 8^*^	*p* = 0.54 (0.08)
	NOR	252 ± 8	342 ± 15^*^	302 ± 15^*^	278 ± 12^*^	285 ± 11^*^	276 ± 10	275 ± 11	*p* < 0.01 (0.73), *p* = 0.51 (0.05)
${\dot{V}\mathrm{CO}}_{2}$ (ml/min)	HYPO	212 ± 9	232 ± 7^*^	208 ± 11	206 ± 10	201 ± 8	219 ± 7	210 ± 7	*p* = 0.53 (0.07)
	NOR	204 ± 11	234 ± 12^*^	194 ± 10	193 ± 12	201 ± 8	200 ± 6	202 ± 8	*p* < 0.01 (0.39), *p* = 0.21 (0.17)
RER	HYPO	0.83 ± 0.01	0.68 ± 0.02^*^	0.70 ± 0.02^*^	0.71 ± 0.01^*^	0.70 ± 0.01^*^	0.75 ± 0.01^*^	0.74 ± 0.01^*^	*p* = 0.59 (0.07)
	NOR	0.81 ± 0.04	0.68 ± 0.02^*^	0.65 ± 0.03^*^	0.70 ± 0.04^*^	0.70 ± 0.02^*^	0.72 ± 0.02	0.73 ± 0.01	*p* < 0.01 (0.53), *p* = 0.29 (0.12)
$\dot{V}E$ (L/min)	HYPO	8.8 ± 0.2	14.3 ± 1.1^*^	10.6 ± 0.5^†^	10.8 ± 0.4^*^^,^^†^	10.3 ± 0.4	11.0 ± 0.3^*^^,^^†^	10.6 ± 0.5^†^	*p* = 0.18 (0.17)
	NOR	8.9 ± 0.5	12.9 ± 0.8^*^	9.1 ± 0.4	9.2 ± 0.6	9.3 ± 0.5	9.8 ± 0.4	9.0 ± 0.5	*p* < 0.01 (0.70), *p* < 0.01 (0.67)
	Pre		30–60 min	60–90 min	90–120 min	120–150 min	150–180 min	
Energy expenditure (kcal∙30 min)	HYPO	36.7 ± 1.4		44.4 ± 1.7^*^	41.0 ± 1.8^*^	40.3 ± 1.5^*^	40.6 ± 1.2^*^	40.5 ± 1.0^*^	*p* = 0.64 (0.07)
	NOR	36.2 ± 1.2		44.8 ± 2.0^*^	40.3 ± 1.6^*^	39.4 ± 1.5	39.4 ± 1.3	38.9 ± 1.3	*p* < 0.01 (0.66), *p* = 0.39 (0.08)

*Values are shown as mean ± SE*.

*The collected data for ${\dot{V}O}_{2}$,${\dot{V}\mathrm{CO}}_{2}$, RER, and $\dot{V}E$ were averaged during 5 min in each point. Energy expenditure for 30 min was calculated using ${\dot{V}O}_{2}$ and ${\dot{V}\mathrm{CO}}_{2}$*.

* *p < 0.05 vs. Pre*.

† *p < 0.05 vs. NOR trial*.

### Urine 8-OHdG Excretion

Urine 8-OHdG excretion did not differ significantly between the two trials (HYPO, 556.5 ± 75.7 ng/ml∙3 h; NOR, 486.8 ± 88.8 ng/ml∙3 h; *p* = 0.37, *d* = 0.27).

### Subjective Variables

Regarding the subjective variables, significant main effects for time (*p* < 0.01, $\eta_{p}^{2}$ = 0.92) and trial (*p* = 0.03, $\eta_{p}^{2}$ = 0.42) were detected for respiratory fatigue. However, there was no significant interaction between time and trial (*p* = 0.30, $\eta_{p}^{2}$ = 0.13). The HYPO trial showed a significantly higher score of respiratory fatigue than the NOR trial immediately after set 3 (*p* < 0.05). The lower-limb muscular fatigue showed a significant interaction between time and trial (*p* < 0.01, $\eta_{p}^{2}$ = 0.31) and a main effect for time (*p* < 0.01, $\eta_{p}^{2}$ = 0.90), but there was no significant main effect for trial (*p* = 0.46, $\eta_{p}^{2}$ = 0.06).

## Discussion

The present study was designed to determine the effects of acute repeated-sprint exercise in moderate hypoxia on exercise-induced cytokines (i.e., IL-6 and IL-1ra), muscle damage (i.e., Mb) and oxidative stress markers (i.e., LPO and 8-OHdG), and angiogenic growth factor (i.e., VEGF) responses among athletes. The novel finding of the present study was that acute repeated-sprint exercise in moderate hypoxia did not exaggerate the exercise-induced cytokines, muscle damage and oxidative stress markers, and angiogenic growth factor responses than those elicited by the same exercise performed in normoxia.

### Inflammatory and Muscle Damage Responses

The exercise-induced blood lactate elevation was greater in hypoxia than in normoxia, although it did not affect significantly power output during exercise. Because ${\dot{V}O}_{2}$ during exercise was significantly lower in the HYPO trial, it appears that anaerobic metabolism (i.e., the glycolytic pathway) was promoted to compensate for impaired aerobic metabolism. Indeed, previous studies have suggested that the contribution of anaerobic energy supply during high-intensity exercise was augmented in hypoxia (Ogura et al., 2006; Bowtell et al., 2014). We hypothesized that the promoted glycolytic pathway during acute repeated-sprint exercise in hypoxia would elicit exercise-induced IL-6 elevation, followed by an increase in the anti-inflammatory response (i.e., IL-1ra). However, this hypothesis was not supported, as both trials showed similar increases in IL-6 and IL-1ra during 3 h of post-exercise. In the present study, the post-exercise IL-6 concentration was relatively low (HYPO, 2.19 ± 0.31 pg/ml; NOR, 2.72 ± 0.60 pg/ml), but the majority of previous studies using prolonged exercise (e.g., approximately 2 h of running) reported a marked increase in IL-6 (before, 0.88 ± 0.16 pg/ml; after, 9.37 ± 1.67 pg/ml) (Nieman et al., 2015). Moreover, the prolonged exercise-induced elevation of IL-6 elicited a subsequent increase in IL-1ra concentrations (Ronsen et al., 2002). Therefore, a smaller elevation of IL-6 would explain the lack of an increase in IL-1ra after exercise. Goods et al. (2016) demonstrated that the IL-6 response to repeated-cycle sprint exercise in moderate hypoxia (three successive sets of 9 s × 5 s maximal sprints; F_i_O_2_, 14.5%) did not differ significantly compared with the same exercise in normoxia. Thus, the short duration of exercise (i.e., low energy expenditure) would not be sufficient to stimulate IL-6 elevation.

The average power output during sprint exercise did not differ significantly between the two trials. When prolonged running exercise in hypoxia was conducted at the same relative exercise intensity than normoxia (running velocity was lower in hypoxia than in normoxia), exercise-induced Mb elevation was significantly lower in hypoxia than in normoxia (Sumi et al., 2018). In contrast, the present study did not reveal a significant difference in exercise-induced Mb elevation between trials, suggesting that repeated-cycling exercise (short duration of exercise) in moderate hypoxia did not trigger a further increase in muscle damage markers compared with the same exercise in normoxia. However, because exercise using a cycle ergometer mainly consisting of concentric muscle contraction, exercise-induced Mb elevation was relatively lower than that reported in previous studies using running exercise, which involves eccentric muscle contraction (Nieman et al., 2018; Sumi et al., 2018). Moreover, exercise mode (running or cycling) might be one of the factors supporting elevation for shear stress. Further studies are therefore required using different exercise modes (running sprint or shuttle sprint).

### Oxidative Stress

Exercise elicits the production of reactive oxygen species, resulting in increased oxidative stress (Møller et al., 1996). In addition, exercise-induced oxidative stress in hypoxia was augmented despite lower ${\dot{V}O}_{2}$ during exercise (Bailey et al., 2001). Several previous studies have reported increased oxidative stress during exposure to hypoxia (Pialoux et al., 2009; Quindry et al., 2016), and maximal sprint exercise in severe hypoxia (F_i_O_2_, 10.4%) also augmented the exercise-induced elevation of oxidative stress markers (Morales-Alamo et al., 2012). Among several types of oxidative stress markers (indicators of lipid, protein, and DNA damage), lipid peroxidation and DNA damage were previously shown to be elevated during the early phase after exercise (Morales-Alamo and Calbet, 2014; Pingitore et al., 2015). Therefore, we hypothesized that acute repeated-sprint exercise in hypoxia would increase post-exercise oxidative stress concentrations. However, no significant differences in oxidative stress markers (i.e., LPO and 8-OHdG) were found between the two trials. Goods et al. (2016) indicated that repeated-sprint exercise in hypoxia (F_i_O_2_, 14.5%) did not cause a further elevation in F_2_-isoprostane (an oxidative stress marker) compared with the same exercise in normoxia. Collectively, acute repeated-sprint exercise in hypoxia would not aggravate oxidative stress, at least the exercise in moderate hypoxia and cycling mode. However, because the present subjects were well-trained athletes, they may be highly resistant to oxidative stress due to superior antioxidant capacity, as suggested in a previous study (Pialoux et al., 2006). Further investigations using different altitude levels, and with various types of athletes (i.e., team sports, racquet sports) and in the subjects with different training backgrounds (recreational, highly-trained, elite athletes) would be required.

### Angiogenic Growth Factors

Endurance training induces skeletal muscle adaptations, including an increase in capillary density (angiogenesis) (Baum et al., 2015). Particularly, prolonged exercise in hypoxia increases serum VEGF (Wahl et al., 2013), which is mediated by the upregulation of hypoxia inducible factor-1 alpha, and contributes to augmented angiogenic adaptation (Lundby et al., 2009). Moreover, increased capillary networks around muscle fibers with improved O_2_ supply would attenuate the power output decrement during repeated-sprint exercise, because muscle O_2_ uptake between rest periods is associated with repeated-sprint performance (Girard et al., 2011; Billaut et al., 2012; Mendez-Villanueva et al., 2012). We hypothesized that maximal sprint exercise in hypoxia would elicit a VEGF response during post-exercise. To our knowledge, this is the first report to present an acute VEGF response following repeated-sprint exercise in hypoxia. Wahl et al. (2011) demonstrated that high-intensity exercise (4 s × 30 s maximal sprints) increased the serum VEGF concentration at 10 min after exercise, but it rapidly returned to the baseline level during 60 min after exercise. In the present study, acute repeated-sprint exercise in hypoxia did not promote the VEGF response. The lack of exercise-induced VEGF elevation could be partly explained by the insufficient exercise duration and/or the duration of hypoxic exposure. With regard to training adaptation, 4 weeks of RSH did not change VEGF mRNA expression in muscle (Faiss et al., 2013b). However, there was a significant increase in VEGF mRNA following combined treatments with 2 weeks of RSH and chronic hypoxic exposure (14 h/day) (Brocherie et al., 2018). In addition, the elevation of VEGF may be dependent on the level of hypoxia. According to a previous study (Wahl et al., 2013), endurance exercise in severe hypoxia (F_i_O_2_; 13.2%) showed a substantial increase in serum VEGF, whereas the increase was disappeared after exercising in mild hypoxia (F_i_O_2_; 15.9%) or normoxia. Moreover, according to a previous study (Kraus et al., 2004), there was a large individual variation in time course of plasma VEGF response. In addition, the correlation between VEGF mRNA and protein in muscle tissue was relatively minor (Kraus et al., 2004). Therefore, the determination of plasma VEGF alone might be insufficient to draw robust conclusion for angiogenesis.

### Resting Energy Expenditure During the Post-Exercise Period

High-intensity exercise augmented resting energy expenditure during the post-exercise period and it is suggested to play a role in weight management (Schubert et al., 2017). A previous study indicated that elevated resting energy expenditure was maintained for several hours following maximal sprint exercise (6 s × 30 s maximal sprints) (Tucker et al., 2016). Increased post-exercise energy expenditure is caused by several factors, including augmented glycogen resynthesis, lipolysis, and muscle-protein turnover (Borsheim and Bahr, 2003). In the present study, it was notable that the HYPO trial augmented post-exercise energy expenditure for a longer period than the NOR trial. Considering that accumulated metabolites (e.g., lactate and pH) due to exercise contribute to the post-exercise increase in oxygen consumption (Cheetham et al., 1986; Hargreaves et al., 1998), the higher blood lactate concentration after exercise in the HYPO trial may partly explain the sustained increase in resting energy expenditure during the post-exercise period. From a different perspective as health promotion, several previous studies indicated that exercise in hypoxia would promote physical well-being among elderly people (Millet et al., 2016; Girard et al., 2017b; Camacho-Cardenosa et al., 2018). Furthermore, augmented post-exercise energy expenditure in hypoxia might help to improve weight management in obese individual (Girard et al., 2017b).

### Practical Application

From a practical standpoint, the present results suggest that concerns about negative impacts (e.g., excessive fatigue or inflammatory responses) after repeated-sprint exercise in hypoxia are not necessary among athletes. In support of this idea, Richardson et al. (2016) indicated that 2 weeks of sprint training in hypoxia (six sessions in total) did not exacerbate plasma IL-6 or tumor necrosis factor-alpha (TNF-α) concentrations. Furthermore, six training sessions for RSH (double-poling exercise) did not elicit further increases in mucosal immune function (Immunoglobulin A) than in the same training in normoxia (Born et al., 2016). Moreover, the scores of perceptual discomfort (overall peripheral discomfort, difficulty breathing, and lower-limb discomfort) were improved during RSH (Brocherie et al., 2017). However, athletes may need to take caution when RSH is conducted frequently during a short period (e.g., training twice a day or successive days of training) which is utilized by Kasai et al. (2017, 2019).

The influence of augmented inflammatory and oxidative stress on training adaptations in muscle is still under debate, and some reports suggested that these factors could be positive (Niess et al., 2003). On the other hand, excessive reactive oxygen species and inflammatory response also lead to aggravated muscle fatigue and dysfunction (Zuo and Pannell, 2015; Duchesne et al., 2017). When the athletes perform RSH in addition to their daily training program, there would be several concerns for performance decrement because of increase in inflammatory and oxidative stress responses. Therefore, the present findings with presenting no apparent impact for hypoxia-induced inflammatory and oxidative stress responses would be beneficial in terms of maintaining physical condition to avoid the risk of overreaching/overtraining in athletes.

## Conclusion

Inflammatory, muscle damage, oxidative stress, and angiogenic growth factor responses to repeated-sprint exercise did not differ in hypoxia and normoxia.

## Data Availability

All datasets generated for this study are included in the manuscript and/or the supplementary files.

## Ethics Statement

Ethics Committee for Human Experiments at Ritsumeikan University.

## Author Contributions

NK and KG contributed to experimental design. NK, CK, DS, and AI performed data collection. NK performed the statistical analysis, interpretation of data, and drafting of the paper. KG revised the work and final approval of the manuscript. All co-authors read and approved the final manuscript.

### Conflict of Interest Statement

The authors declare that the research was conducted in the absence of any commercial or financial relationships that could be construed as a potential conflict of interest.
